# Supplementary material for: Tracking cells in epithelial acini by light sheet microscopy reveals proximity effects in breast cancer initiation
Source: eLife. 2020 Jul 21;9:e54066. doi: 10.7554/eLife.54066 (PMC7373425; doi:10.7554/eLife.54066)
Supplement: Supplementary file 1. [file elife-54066-supp1.zip › Supplement_File_1.html]

Organoid tumors


Code 

- Show All Code
- Hide All Code
- Download Rmd

# Organoid tumors

Random cells in organoids are transduced to express oncogenes. However, only some transduced cells proliferate and lead to tumor formation. Initial observations suggest that tumors originate from clusters of transduced cells.

Goal: Find which features of transduced cell clusters best predict the formation of tumors.

For each cluster of transduced cells, the features to test are: - number of cells in the organoid - ratio of number of cells to organoid surface area (~cell density) - number of transduced cells in the organoid - number of cells in the cluster volume - number of transduced cells in the cluster - average pairwise distance between all cells in the cluster volume - average pairwise distance between transduced cells in the cluster - fraction of transduced cells in the cluster volume - number of contacts between transduced cells in the cluster

#### Load the data

Data consists of two csv files per organoid at the start of the experiment: - one file containing x,y,z coordinates and ID of all cells in the organoid, - one file containing IDs of all transduced cells in the organoid with 1 in the second column indicating that the cluster the transduced cell belongs to will produce a tumor. Only one transduced cell per cluster is annotated.


```
data.dir <- "organoids_tumors"
file.list <- list.files(data.dir, pattern = "Pos", full.names = TRUE)
organoids <- list()
for(i in 1:length(file.list)){
  # Read cells positions
  # The files have 2 lines above the header (hence the use of skip=2)
  # Be careful: sometimes a third empty line is at the top of the file and should be removed before reading here
  # Also read.csv() issues a warning if the file doesn't end with an empty line
  cells <- read.csv(file.list[[i]], header=TRUE, sep=",",  stringsAsFactors = FALSE, skip = 2)
  # Remove unnecessary columns
  cells <- cells[, -c(4:9)]
  # Rename columns
  colnames(cells) <- c("x","y","z","id")
  # Read labels
  annotation.file <- gsub("Pos", "Label", file.list[[i]])
  annotations <- read.csv(annotation.file, header=TRUE, sep=",", stringsAsFactors = FALSE)
  annotations <- cbind(annotations, 1)
  colnames(annotations) <- c("id", "tumor", "transduced")
  # Combine positions and annotations based on cell id
  cells <- merge(cells, annotations, by = "id", all.x = TRUE)
  cells[is.na(cells)] <- 0
  organoids[[i]] <- cells
}
```


```
incomplete final line found by readTableHeader on 'organoids_tumors/Label_0010.csv'incomplete final line found by readTableHeader on 'organoids_tumors/Label_0018.csv'
```

#### Identify clusters of transduced cells and extract features


```
library(dbscan)
library(car)
library(RColorBrewer)
library(pals)
library(dynamicTreeCut)
library(dendextend)
palette.cells <- rev(colorRampPalette(brewer.pal(11, "Spectral"))(3+1L))
palette.clusters <- alphabet()
palette.clusters[c("damson","violet")] <- palette.clusters[c("violet","damson")]
organoid.features <- data.frame(number.of.cells = numeric(),
                                cell.density = numeric(),
                                number.of.transduced.cells = numeric(), 
                                tumor = numeric())
clusters <- data.frame(id = character(), 
                       number.of.cells.in.organoid = numeric(),
                       organoid.cell.density = numeric(),
                       number.of.transduced.cells.in.organoid = numeric(), 
                       number.of.cells.in.a.cluster = numeric(),
                       number.of.transduced.cells.in.a.cluster = numeric(),
                       average.distance.between.cells.in.a.cluster = numeric(),
                       average.distance.between.transduced.cells.in.a.cluster = numeric(),
                       fraction.of.transduced.cells.in.a.cluster = numeric(),
                       number.of.contacts.between.transduced.cells.in.a.cluster = numeric(),
                       tumor = numeric())
for(i in 1:length(organoids)) {
  cells <- organoids[[i]]
  
  # Coordinates for some organoids seem to be in nm instead of um
  if (max(cells[,c("x","y","z")])>1000) {
    cells[,c("x","y","z")] <- cells[,c("x","y","z")]/1000
  }
  
  # Estimate average cell diameter as average distance between nearest neighbours
  NN.dist <- kNNdist(cells[,c("x","y","z")], k=1)
  avg.cell.diameter <- mean(NN.dist)
  sd.cell.diameter <- sd(NN.dist)
  
  # Remove outliers
  # Outliers are cells that have a very large distance to their nearest neighbour
  idx.outliers <- which(NN.dist>avg.cell.diameter+3*sd.cell.diameter)
  if(length(idx.outliers)>0) {
    cells <- cells[-idx.outliers,]
  }
  
  # Recompute values for the organoid
  NN.dist <- kNNdist(cells[,c("x","y","z")], k=1)
  avg.cell.diameter <- mean(NN.dist)
  sd.cell.diameter <- sd(NN.dist)
  
  # Estimate organoid diameter as distance between the two most distant cells
  org.diameter <- max(dist(cells[,c("x","y","z")]))
  # Cell density is ratio of number of cells to organoid surface area
  # Multiply by 1000 to bring on the same scale as other features
  cell.density <- nrow(cells)/(4*pi*org.diameter^2) * 1000
  
  # # Show positions of cells in organoid
  # scatter3d(cells$x,cells$y,cells$z, surface = FALSE, sphere.size = 3, surface.col = palette.cells, col = palette.cells, xlab="x",ylab="y",zlab="z", axis.scales = FALSE,  axis.col = c("black", "black", "black"), groups = as.factor(cells$transduced+cells$tumor))
  
  # Identify clusters of transduced cells
  transduced.cells <- cells[which(cells$transduced == 1),]
  D <- dist(transduced.cells[,c("x","y","z")])
  n <- nrow(transduced.cells)
  if(n>2) {
    hc <- hclust(D, method = "complete")
    cluster.membership <- cutreeHybrid(hc, minClusterSize = 1, distM = as.matrix(D))
    transduced.cells$cluster <- cluster.membership$labels
    # Show clusters on dendrogram
    dend <- as.dendrogram(hc)
    dend %>% set("leaves_pch", 19-10*transduced.cells$tumor[order.dendrogram(dend)]) %>%  # node type
      set("leaves_cex", 2) %>%  # node size
      set("leaves_col", palette.clusters[transduced.cells$cluster[order.dendrogram(dend)]+1]) %>% # leaves colors
      set("labels", "") %>%
    plot(main = paste0("Clusters of transduced cells in organoid ",i))
    
  } else if(n==2) {
    if(D<avg.cell.diameter+2*sd.cell.diameter) { # Form one cluster
      transduced.cells$cluster <- 1
    } else { # Form two clusters
      transduced.cells$cluster <- c(1,2)
    }
  } else if(n==1) {
    transduced.cells$cluster <- 1
  } else {
    print(paste0("Organoid ",i," has no transduced cell."))
  }
  # Extract cluster features
  for(j in 1:max(transduced.cells$cluster)) {
    cluster.cells <- transduced.cells[transduced.cells$cluster==j,]
    id <- paste0(i,".",j)
    
    # Number of cells in the organoid
    n.organoid <- nrow(cells)
    
    # Number of transduced cells in the organoid
    n.transduced.organoid <- nrow(transduced.cells)
    
    # Number of cells in the cluster volume
    # The cluster volume is defined as the sphere centrered at the centre of mass of the cluster
    # with diameter equal to the distance between the two farthest transduced cells of the cluster
    cluster.centre <- apply(cluster.cells[, c("x","y","z")], 2, mean)
    if (nrow(cluster.cells)>1) {
      dist.cluster.cells <- dist(cluster.cells[, c("x","y","z")])
      cluster.diameter <- max(dist.cluster.cells) + 1e-6 # Add small amount to deal with numerical precision issues
    } else {
      dist.cluster.cells <- 0
      cluster.diameter <- 0
    }
    dist2centre <- apply(cells[, c("x","y","z")], 1, function(x) {dist(rbind(x, cluster.centre))})
    volume.cells <- cells[which(dist2centre<=cluster.diameter), c("x","y","z")] 
    n.cluster <- nrow(volume.cells)
    
    # Number of transduced cells in the cluster
    n.transduced.cluster <- nrow(cluster.cells)
  
    # Average pairwise distance between transduced cells in the cluster
    avg.dist.cluster.cells <- mean(dist.cluster.cells)
    
    # Average pairwise distance between all cells in the cluster
    if (nrow(volume.cells)>1) {
      avg.dist.volume.cells <- mean(dist(volume.cells))
    } else {
      avg.dist.volume.cells <- avg.dist.cluster.cells
    }    
  
    # Number of contacts between transduced cells in the cluster
    # Two cells are presumed in contact if they are less than 1 cell diameter (+ sd) apart 
    n.contacts.transduced <- length(which(dist.cluster.cells<=avg.cell.diameter+2*sd.cell.diameter))
    
    # Fraction of transduced cells in the cluster volume (in percent)
    f.transduced <- 100
    if (n.cluster>0) {
      f.transduced <- n.transduced.cluster/n.cluster * 100
    }
    
    # Does the cluster give rise to a tumor ?
    tumor <- if(sum(cluster.cells$tumor>0)) 1 else 0
    
    df <- data.frame(id = id, 
                     number.of.cells.in.organoid = n.organoid,
                     organoid.cell.density = cell.density,
                     number.of.transduced.cells.in.organoid = n.transduced.organoid, 
                     number.of.cells.in.a.cluster = n.cluster,
                     number.of.transduced.cells.in.a.cluster = n.transduced.cluster,
                     average.distance.between.cells.in.a.cluster = avg.dist.volume.cells,
                     average.distance.between.transduced.cells.in.a.cluster = avg.dist.cluster.cells,
                     fraction.of.transduced.cells.in.a.cluster = f.transduced,
                     number.of.contacts.between.transduced.cells.in.a.cluster = n.contacts.transduced,
                     tumor = tumor)
    
    clusters <- rbind(clusters, df)
    
  }
  org.tumor <- if(sum(cells$tumor>0)) 1 else 0
  organoid.features[i,] <- c(nrow(cells), cell.density, n, org.tumor)
}
```


```
 ..cutHeight not given, setting it to 74.3  ===>  99% of the (truncated) height range in dendro.
 ..done.
```


```
The lengths of the new labels is shorter than the number of leaves in the dendrogram - labels are recycled.
```


```
 ..cutHeight not given, setting it to 57.5  ===>  99% of the (truncated) height range in dendro.
 ..done.
```


```
 ..cutHeight not given, setting it to 76.9  ===>  99% of the (truncated) height range in dendro.
 ..done.
```


```
The lengths of the new labels is shorter than the number of leaves in the dendrogram - labels are recycled.
```


```
 ..cutHeight not given, setting it to 69  ===>  99% of the (truncated) height range in dendro.
 ..done.
```


```
The lengths of the new labels is shorter than the number of leaves in the dendrogram - labels are recycled.
```


```
 ..cutHeight not given, setting it to 67.3  ===>  99% of the (truncated) height range in dendro.
 ..done.
```


```
The lengths of the new labels is shorter than the number of leaves in the dendrogram - labels are recycled.
```


```
 ..cutHeight not given, setting it to 75.9  ===>  99% of the (truncated) height range in dendro.
 ..done.
```


```
The lengths of the new labels is shorter than the number of leaves in the dendrogram - labels are recycled.
```


```
 ..cutHeight not given, setting it to 67.6  ===>  99% of the (truncated) height range in dendro.
 ..done.
```


```
The lengths of the new labels is shorter than the number of leaves in the dendrogram - labels are recycled.
```


```
 ..cutHeight not given, setting it to 65.3  ===>  99% of the (truncated) height range in dendro.
 ..done.
```


```
The lengths of the new labels is shorter than the number of leaves in the dendrogram - labels are recycled.
```


```
 ..cutHeight not given, setting it to 47.3  ===>  99% of the (truncated) height range in dendro.
 ..done.
```


```
The lengths of the new labels is shorter than the number of leaves in the dendrogram - labels are recycled.
```


```
 ..cutHeight not given, setting it to 31.7  ===>  99% of the (truncated) height range in dendro.
 ..done.
```


```
The lengths of the new labels is shorter than the number of leaves in the dendrogram - labels are recycled.
```


```
 ..cutHeight not given, setting it to 52.4  ===>  99% of the (truncated) height range in dendro.
 ..done.
```


```
The lengths of the new labels is shorter than the number of leaves in the dendrogram - labels are recycled.
```


```
 ..cutHeight not given, setting it to 22.6  ===>  99% of the (truncated) height range in dendro.
 ..done.
```


```
The lengths of the new labels is shorter than the number of leaves in the dendrogram - labels are recycled.
```


```
 ..cutHeight not given, setting it to 61.7  ===>  99% of the (truncated) height range in dendro.
 ..done.
```


```
The lengths of the new labels is shorter than the number of leaves in the dendrogram - labels are recycled.
```


```
 ..cutHeight not given, setting it to 25.1  ===>  99% of the (truncated) height range in dendro.
 ..done.
```


```
The lengths of the new labels is shorter than the number of leaves in the dendrogram - labels are recycled.
```


```
 ..cutHeight not given, setting it to 58.5  ===>  99% of the (truncated) height range in dendro.
 ..done.
```


```
The lengths of the new labels is shorter than the number of leaves in the dendrogram - labels are recycled.
```


```
 ..cutHeight not given, setting it to 52.6  ===>  99% of the (truncated) height range in dendro.
 ..done.
```


```
The lengths of the new labels is shorter than the number of leaves in the dendrogram - labels are recycled.
```


```
 ..cutHeight not given, setting it to 57.4  ===>  99% of the (truncated) height range in dendro.
 ..done.
```


```
The lengths of the new labels is shorter than the number of leaves in the dendrogram - labels are recycled.
```

#### Logistic regression with model selection

In this approach, we build a model for each possible combination of features (i.e. 2\*\*9 models for 9 features) and use the Akaike information criterion to select the best model and evaluate the contribution of features retained in this model. Because it is possible the AIC is subject to small variations and because models are considered indistinguishable if the difference in AIC is less than 2, we also look at variable importance over multiple/all models


```
library(glmulti)
```


```
Loading required package: rJava
JavaVM: requested Java version ((null)) not available. Using Java at "" instead.
JavaVM: Failed to load JVM: /bundle/Libraries/libserver.dylib
JavaVM FATAL: Failed to load the jvm library.
Error : .onLoad failed in loadNamespace() for 'glmulti', details:
  call: .jinit(parameters = "-XmX512m", force.init = TRUE)
  error: JNI_GetCreatedJavaVMs returned -1

Error: package or namespace load failed for ‘glmulti’
```

#### Logistic regression with elastic net regularization

This approach starts with all features in the model and tries to set to 0 as many of the coefficients as possible.

LS0tCnRpdGxlOiAiT3JnYW5vaWQgdHVtb3JzIgpvdXRwdXQ6IGh0bWxfbm90ZWJvb2sKLS0tCgpSYW5kb20gY2VsbHMgaW4gb3JnYW5vaWRzIGFyZSB0cmFuc2R1Y2VkIHRvIGV4cHJlc3Mgb25jb2dlbmVzLiBIb3dldmVyLCBvbmx5IHNvbWUgdHJhbnNkdWNlZCBjZWxscyBwcm9saWZlcmF0ZSBhbmQgbGVhZCB0byB0dW1vciBmb3JtYXRpb24uIEluaXRpYWwgb2JzZXJ2YXRpb25zIHN1Z2dlc3QgdGhhdCB0dW1vcnMgb3JpZ2luYXRlIGZyb20gY2x1c3RlcnMgb2YgdHJhbnNkdWNlZCBjZWxscy4gCgpHb2FsOiBGaW5kIHdoaWNoIGZlYXR1cmVzIG9mIHRyYW5zZHVjZWQgY2VsbCBjbHVzdGVycyBiZXN0IHByZWRpY3QgdGhlIGZvcm1hdGlvbiBvZiB0dW1vcnMuCgpGb3IgZWFjaCBjbHVzdGVyIG9mIHRyYW5zZHVjZWQgY2VsbHMsIHRoZSBmZWF0dXJlcyB0byB0ZXN0IGFyZToKLSBudW1iZXIgb2YgY2VsbHMgaW4gdGhlIG9yZ2Fub2lkCi0gcmF0aW8gb2YgbnVtYmVyIG9mIGNlbGxzIHRvIG9yZ2Fub2lkIHN1cmZhY2UgYXJlYSAofmNlbGwgZGVuc2l0eSkKLSBudW1iZXIgb2YgdHJhbnNkdWNlZCBjZWxscyBpbiB0aGUgb3JnYW5vaWQKLSBudW1iZXIgb2YgY2VsbHMgaW4gdGhlIGNsdXN0ZXIgdm9sdW1lCi0gbnVtYmVyIG9mIHRyYW5zZHVjZWQgY2VsbHMgaW4gdGhlIGNsdXN0ZXIKLSBhdmVyYWdlIHBhaXJ3aXNlIGRpc3RhbmNlIGJldHdlZW4gYWxsIGNlbGxzIGluIHRoZSBjbHVzdGVyIHZvbHVtZQotIGF2ZXJhZ2UgcGFpcndpc2UgZGlzdGFuY2UgYmV0d2VlbiB0cmFuc2R1Y2VkIGNlbGxzIGluIHRoZSBjbHVzdGVyCi0gZnJhY3Rpb24gb2YgdHJhbnNkdWNlZCBjZWxscyBpbiB0aGUgY2x1c3RlciB2b2x1bWUKLSBudW1iZXIgb2YgY29udGFjdHMgYmV0d2VlbiB0cmFuc2R1Y2VkIGNlbGxzIGluIHRoZSBjbHVzdGVyCgoKIyMjIyBMb2FkIHRoZSBkYXRhCkRhdGEgY29uc2lzdHMgb2YgdHdvIGNzdiBmaWxlcyBwZXIgb3JnYW5vaWQgYXQgdGhlIHN0YXJ0IG9mIHRoZSBleHBlcmltZW50OgotIG9uZSBmaWxlIGNvbnRhaW5pbmcgeCx5LHogY29vcmRpbmF0ZXMgYW5kIElEIG9mIGFsbCBjZWxscyBpbiB0aGUgb3JnYW5vaWQsCi0gb25lIGZpbGUgY29udGFpbmluZyBJRHMgb2YgYWxsIHRyYW5zZHVjZWQgY2VsbHMgaW4gdGhlIG9yZ2Fub2lkIHdpdGggMSBpbiB0aGUgc2Vjb25kIGNvbHVtbiBpbmRpY2F0aW5nIHRoYXQgdGhlIGNsdXN0ZXIgdGhlIHRyYW5zZHVjZWQgY2VsbCBiZWxvbmdzIHRvIHdpbGwgcHJvZHVjZSBhIHR1bW9yLiBPbmx5IG9uZSB0cmFuc2R1Y2VkIGNlbGwgcGVyIGNsdXN0ZXIgaXMgYW5ub3RhdGVkLgpgYGB7cn0KCmRhdGEuZGlyIDwtICJvcmdhbm9pZHNfdHVtb3JzIgoKZmlsZS5saXN0IDwtIGxpc3QuZmlsZXMoZGF0YS5kaXIsIHBhdHRlcm4gPSAiUG9zIiwgZnVsbC5uYW1lcyA9IFRSVUUpCgpvcmdhbm9pZHMgPC0gbGlzdCgpCmZvcihpIGluIDE6bGVuZ3RoKGZpbGUubGlzdCkpewogICMgUmVhZCBjZWxscyBwb3NpdGlvbnMKICAjIFRoZSBmaWxlcyBoYXZlIDIgbGluZXMgYWJvdmUgdGhlIGhlYWRlciAoaGVuY2UgdGhlIHVzZSBvZiBza2lwPTIpCiAgIyBCZSBjYXJlZnVsOiBzb21ldGltZXMgYSB0aGlyZCBlbXB0eSBsaW5lIGlzIGF0IHRoZSB0b3Agb2YgdGhlIGZpbGUgYW5kIHNob3VsZCBiZSByZW1vdmVkIGJlZm9yZSByZWFkaW5nIGhlcmUKICAjIEFsc28gcmVhZC5jc3YoKSBpc3N1ZXMgYSB3YXJuaW5nIGlmIHRoZSBmaWxlIGRvZXNuJ3QgZW5kIHdpdGggYW4gZW1wdHkgbGluZQogIGNlbGxzIDwtIHJlYWQuY3N2KGZpbGUubGlzdFtbaV1dLCBoZWFkZXI9VFJVRSwgc2VwPSIsIiwgIHN0cmluZ3NBc0ZhY3RvcnMgPSBGQUxTRSwgc2tpcCA9IDIpCiAgIyBSZW1vdmUgdW5uZWNlc3NhcnkgY29sdW1ucwogIGNlbGxzIDwtIGNlbGxzWywgLWMoNDo5KV0KICAjIFJlbmFtZSBjb2x1bW5zCiAgY29sbmFtZXMoY2VsbHMpIDwtIGMoIngiLCJ5IiwieiIsImlkIikKICAjIFJlYWQgbGFiZWxzCiAgYW5ub3RhdGlvbi5maWxlIDwtIGdzdWIoIlBvcyIsICJMYWJlbCIsIGZpbGUubGlzdFtbaV1dKQogIGFubm90YXRpb25zIDwtIHJlYWQuY3N2KGFubm90YXRpb24uZmlsZSwgaGVhZGVyPVRSVUUsIHNlcD0iLCIsIHN0cmluZ3NBc0ZhY3RvcnMgPSBGQUxTRSkKICBhbm5vdGF0aW9ucyA8LSBjYmluZChhbm5vdGF0aW9ucywgMSkKICBjb2xuYW1lcyhhbm5vdGF0aW9ucykgPC0gYygiaWQiLCAidHVtb3IiLCAidHJhbnNkdWNlZCIpCiAgIyBDb21iaW5lIHBvc2l0aW9ucyBhbmQgYW5ub3RhdGlvbnMgYmFzZWQgb24gY2VsbCBpZAogIGNlbGxzIDwtIG1lcmdlKGNlbGxzLCBhbm5vdGF0aW9ucywgYnkgPSAiaWQiLCBhbGwueCA9IFRSVUUpCiAgY2VsbHNbaXMubmEoY2VsbHMpXSA8LSAwCiAgb3JnYW5vaWRzW1tpXV0gPC0gY2VsbHMKfQpgYGAKCiMjIyMgSWRlbnRpZnkgY2x1c3RlcnMgb2YgdHJhbnNkdWNlZCBjZWxscyBhbmQgZXh0cmFjdCBmZWF0dXJlcwpgYGB7cn0KbGlicmFyeShkYnNjYW4pCmxpYnJhcnkoY2FyKQpsaWJyYXJ5KFJDb2xvckJyZXdlcikKbGlicmFyeShwYWxzKQpsaWJyYXJ5KGR5bmFtaWNUcmVlQ3V0KQpsaWJyYXJ5KGRlbmRleHRlbmQpCgpwYWxldHRlLmNlbGxzIDwtIHJldihjb2xvclJhbXBQYWxldHRlKGJyZXdlci5wYWwoMTEsICJTcGVjdHJhbCIpKSgzKzFMKSkKcGFsZXR0ZS5jbHVzdGVycyA8LSBhbHBoYWJldCgpCnBhbGV0dGUuY2x1c3RlcnNbYygiZGFtc29uIiwidmlvbGV0IildIDwtIHBhbGV0dGUuY2x1c3RlcnNbYygidmlvbGV0IiwiZGFtc29uIildCgpvcmdhbm9pZC5mZWF0dXJlcyA8LSBkYXRhLmZyYW1lKG51bWJlci5vZi5jZWxscyA9IG51bWVyaWMoKSwKICAgICAgICAgICAgICAgICAgICAgICAgICAgICAgICBjZWxsLmRlbnNpdHkgPSBudW1lcmljKCksCiAgICAgICAgICAgICAgICAgICAgICAgICAgICAgICAgbnVtYmVyLm9mLnRyYW5zZHVjZWQuY2VsbHMgPSBudW1lcmljKCksIAogICAgICAgICAgICAgICAgICAgICAgICAgICAgICAgIHR1bW9yID0gbnVtZXJpYygpKQoKY2x1c3RlcnMgPC0gZGF0YS5mcmFtZShpZCA9IGNoYXJhY3RlcigpLCAKICAgICAgICAgICAgICAgICAgICAgICBudW1iZXIub2YuY2VsbHMuaW4ub3JnYW5vaWQgPSBudW1lcmljKCksCiAgICAgICAgICAgICAgICAgICAgICAgb3JnYW5vaWQuY2VsbC5kZW5zaXR5ID0gbnVtZXJpYygpLAogICAgICAgICAgICAgICAgICAgICAgIG51bWJlci5vZi50cmFuc2R1Y2VkLmNlbGxzLmluLm9yZ2Fub2lkID0gbnVtZXJpYygpLCAKICAgICAgICAgICAgICAgICAgICAgICBudW1iZXIub2YuY2VsbHMuaW4uYS5jbHVzdGVyID0gbnVtZXJpYygpLAogICAgICAgICAgICAgICAgICAgICAgIG51bWJlci5vZi50cmFuc2R1Y2VkLmNlbGxzLmluLmEuY2x1c3RlciA9IG51bWVyaWMoKSwKICAgICAgICAgICAgICAgICAgICAgICBhdmVyYWdlLmRpc3RhbmNlLmJldHdlZW4uY2VsbHMuaW4uYS5jbHVzdGVyID0gbnVtZXJpYygpLAogICAgICAgICAgICAgICAgICAgICAgIGF2ZXJhZ2UuZGlzdGFuY2UuYmV0d2Vlbi50cmFuc2R1Y2VkLmNlbGxzLmluLmEuY2x1c3RlciA9IG51bWVyaWMoKSwKICAgICAgICAgICAgICAgICAgICAgICBmcmFjdGlvbi5vZi50cmFuc2R1Y2VkLmNlbGxzLmluLmEuY2x1c3RlciA9IG51bWVyaWMoKSwKICAgICAgICAgICAgICAgICAgICAgICBudW1iZXIub2YuY29udGFjdHMuYmV0d2Vlbi50cmFuc2R1Y2VkLmNlbGxzLmluLmEuY2x1c3RlciA9IG51bWVyaWMoKSwKICAgICAgICAgICAgICAgICAgICAgICB0dW1vciA9IG51bWVyaWMoKSkKZm9yKGkgaW4gMTpsZW5ndGgob3JnYW5vaWRzKSkgewogIGNlbGxzIDwtIG9yZ2Fub2lkc1tbaV1dCiAgCiAgIyBDb29yZGluYXRlcyBmb3Igc29tZSBvcmdhbm9pZHMgc2VlbSB0byBiZSBpbiBubSBpbnN0ZWFkIG9mIHVtCiAgaWYgKG1heChjZWxsc1ssYygieCIsInkiLCJ6IildKT4xMDAwKSB7CiAgICBjZWxsc1ssYygieCIsInkiLCJ6IildIDwtIGNlbGxzWyxjKCJ4IiwieSIsInoiKV0vMTAwMAogIH0KICAKICAjIEVzdGltYXRlIGF2ZXJhZ2UgY2VsbCBkaWFtZXRlciBhcyBhdmVyYWdlIGRpc3RhbmNlIGJldHdlZW4gbmVhcmVzdCBuZWlnaGJvdXJzCiAgTk4uZGlzdCA8LSBrTk5kaXN0KGNlbGxzWyxjKCJ4IiwieSIsInoiKV0sIGs9MSkKICBhdmcuY2VsbC5kaWFtZXRlciA8LSBtZWFuKE5OLmRpc3QpCiAgc2QuY2VsbC5kaWFtZXRlciA8LSBzZChOTi5kaXN0KQogIAogICMgUmVtb3ZlIG91dGxpZXJzCiAgIyBPdXRsaWVycyBhcmUgY2VsbHMgdGhhdCBoYXZlIGEgdmVyeSBsYXJnZSBkaXN0YW5jZSB0byB0aGVpciBuZWFyZXN0IG5laWdoYm91cgogIGlkeC5vdXRsaWVycyA8LSB3aGljaChOTi5kaXN0PmF2Zy5jZWxsLmRpYW1ldGVyKzMqc2QuY2VsbC5kaWFtZXRlcikKICBpZihsZW5ndGgoaWR4Lm91dGxpZXJzKT4wKSB7CiAgICBjZWxscyA8LSBjZWxsc1staWR4Lm91dGxpZXJzLF0KICB9CiAgCiAgIyBSZWNvbXB1dGUgdmFsdWVzIGZvciB0aGUgb3JnYW5vaWQKICBOTi5kaXN0IDwtIGtOTmRpc3QoY2VsbHNbLGMoIngiLCJ5IiwieiIpXSwgaz0xKQogIGF2Zy5jZWxsLmRpYW1ldGVyIDwtIG1lYW4oTk4uZGlzdCkKICBzZC5jZWxsLmRpYW1ldGVyIDwtIHNkKE5OLmRpc3QpCiAgCiAgIyBFc3RpbWF0ZSBvcmdhbm9pZCBkaWFtZXRlciBhcyBkaXN0YW5jZSBiZXR3ZWVuIHRoZSB0d28gbW9zdCBkaXN0YW50IGNlbGxzCiAgb3JnLmRpYW1ldGVyIDwtIG1heChkaXN0KGNlbGxzWyxjKCJ4IiwieSIsInoiKV0pKQogICMgQ2VsbCBkZW5zaXR5IGlzIHJhdGlvIG9mIG51bWJlciBvZiBjZWxscyB0byBvcmdhbm9pZCBzdXJmYWNlIGFyZWEKICAjIE11bHRpcGx5IGJ5IDEwMDAgdG8gYnJpbmcgb24gdGhlIHNhbWUgc2NhbGUgYXMgb3RoZXIgZmVhdHVyZXMKICBjZWxsLmRlbnNpdHkgPC0gbnJvdyhjZWxscykvKDQqcGkqb3JnLmRpYW1ldGVyXjIpICogMTAwMAogIAogICMgIyBTaG93IHBvc2l0aW9ucyBvZiBjZWxscyBpbiBvcmdhbm9pZAogICMgc2NhdHRlcjNkKGNlbGxzJHgsY2VsbHMkeSxjZWxscyR6LCBzdXJmYWNlID0gRkFMU0UsIHNwaGVyZS5zaXplID0gMywgc3VyZmFjZS5jb2wgPSBwYWxldHRlLmNlbGxzLCBjb2wgPSBwYWxldHRlLmNlbGxzLCB4bGFiPSJ4Iix5bGFiPSJ5Iix6bGFiPSJ6IiwgYXhpcy5zY2FsZXMgPSBGQUxTRSwgIGF4aXMuY29sID0gYygiYmxhY2siLCAiYmxhY2siLCAiYmxhY2siKSwgZ3JvdXBzID0gYXMuZmFjdG9yKGNlbGxzJHRyYW5zZHVjZWQrY2VsbHMkdHVtb3IpKQogIAogICMgSWRlbnRpZnkgY2x1c3RlcnMgb2YgdHJhbnNkdWNlZCBjZWxscwogIHRyYW5zZHVjZWQuY2VsbHMgPC0gY2VsbHNbd2hpY2goY2VsbHMkdHJhbnNkdWNlZCA9PSAxKSxdCiAgRCA8LSBkaXN0KHRyYW5zZHVjZWQuY2VsbHNbLGMoIngiLCJ5IiwieiIpXSkKICBuIDwtIG5yb3codHJhbnNkdWNlZC5jZWxscykKICBpZihuPjIpIHsKICAgIGhjIDwtIGhjbHVzdChELCBtZXRob2QgPSAiY29tcGxldGUiKQogICAgY2x1c3Rlci5tZW1iZXJzaGlwIDwtIGN1dHJlZUh5YnJpZChoYywgbWluQ2x1c3RlclNpemUgPSAxLCBkaXN0TSA9IGFzLm1hdHJpeChEKSkKICAgIHRyYW5zZHVjZWQuY2VsbHMkY2x1c3RlciA8LSBjbHVzdGVyLm1lbWJlcnNoaXAkbGFiZWxzCiAgICAjIFNob3cgY2x1c3RlcnMgb24gZGVuZHJvZ3JhbQogICAgZGVuZCA8LSBhcy5kZW5kcm9ncmFtKGhjKQogICAgZGVuZCAlPiUgc2V0KCJsZWF2ZXNfcGNoIiwgMTktMTAqdHJhbnNkdWNlZC5jZWxscyR0dW1vcltvcmRlci5kZW5kcm9ncmFtKGRlbmQpXSkgJT4lICAjIG5vZGUgdHlwZQogICAgICBzZXQoImxlYXZlc19jZXgiLCAyKSAlPiUgICMgbm9kZSBzaXplCiAgICAgIHNldCgibGVhdmVzX2NvbCIsIHBhbGV0dGUuY2x1c3RlcnNbdHJhbnNkdWNlZC5jZWxscyRjbHVzdGVyW29yZGVyLmRlbmRyb2dyYW0oZGVuZCldKzFdKSAlPiUgIyBsZWF2ZXMgY29sb3JzCiAgICAgIHNldCgibGFiZWxzIiwgIiIpICU+JQogICAgcGxvdChtYWluID0gcGFzdGUwKCJDbHVzdGVycyBvZiB0cmFuc2R1Y2VkIGNlbGxzIGluIG9yZ2Fub2lkICIsaSkpCiAgICAKICB9IGVsc2UgaWYobj09MikgewogICAgaWYoRDxhdmcuY2VsbC5kaWFtZXRlcisyKnNkLmNlbGwuZGlhbWV0ZXIpIHsgIyBGb3JtIG9uZSBjbHVzdGVyCiAgICAgIHRyYW5zZHVjZWQuY2VsbHMkY2x1c3RlciA8LSAxCiAgICB9IGVsc2UgeyAjIEZvcm0gdHdvIGNsdXN0ZXJzCiAgICAgIHRyYW5zZHVjZWQuY2VsbHMkY2x1c3RlciA8LSBjKDEsMikKICAgIH0KICB9IGVsc2UgaWYobj09MSkgewogICAgdHJhbnNkdWNlZC5jZWxscyRjbHVzdGVyIDwtIDEKICB9IGVsc2UgewogICAgcHJpbnQocGFzdGUwKCJPcmdhbm9pZCAiLGksIiBoYXMgbm8gdHJhbnNkdWNlZCBjZWxsLiIpKQogIH0KICAjIEV4dHJhY3QgY2x1c3RlciBmZWF0dXJlcwogIGZvcihqIGluIDE6bWF4KHRyYW5zZHVjZWQuY2VsbHMkY2x1c3RlcikpIHsKICAgIGNsdXN0ZXIuY2VsbHMgPC0gdHJhbnNkdWNlZC5jZWxsc1t0cmFuc2R1Y2VkLmNlbGxzJGNsdXN0ZXI9PWosXQogICAgaWQgPC0gcGFzdGUwKGksIi4iLGopCiAgICAKICAgICMgTnVtYmVyIG9mIGNlbGxzIGluIHRoZSBvcmdhbm9pZAogICAgbi5vcmdhbm9pZCA8LSBucm93KGNlbGxzKQogICAgCiAgICAjIE51bWJlciBvZiB0cmFuc2R1Y2VkIGNlbGxzIGluIHRoZSBvcmdhbm9pZAogICAgbi50cmFuc2R1Y2VkLm9yZ2Fub2lkIDwtIG5yb3codHJhbnNkdWNlZC5jZWxscykKICAgIAogICAgIyBOdW1iZXIgb2YgY2VsbHMgaW4gdGhlIGNsdXN0ZXIgdm9sdW1lCiAgICAjIFRoZSBjbHVzdGVyIHZvbHVtZSBpcyBkZWZpbmVkIGFzIHRoZSBzcGhlcmUgY2VudHJlcmVkIGF0IHRoZSBjZW50cmUgb2YgbWFzcyBvZiB0aGUgY2x1c3RlcgogICAgIyB3aXRoIGRpYW1ldGVyIGVxdWFsIHRvIHRoZSBkaXN0YW5jZSBiZXR3ZWVuIHRoZSB0d28gZmFydGhlc3QgdHJhbnNkdWNlZCBjZWxscyBvZiB0aGUgY2x1c3RlcgogICAgY2x1c3Rlci5jZW50cmUgPC0gYXBwbHkoY2x1c3Rlci5jZWxsc1ssIGMoIngiLCJ5IiwieiIpXSwgMiwgbWVhbikKICAgIGlmIChucm93KGNsdXN0ZXIuY2VsbHMpPjEpIHsKICAgICAgZGlzdC5jbHVzdGVyLmNlbGxzIDwtIGRpc3QoY2x1c3Rlci5jZWxsc1ssIGMoIngiLCJ5IiwieiIpXSkKICAgICAgY2x1c3Rlci5kaWFtZXRlciA8LSBtYXgoZGlzdC5jbHVzdGVyLmNlbGxzKSArIDFlLTYgIyBBZGQgc21hbGwgYW1vdW50IHRvIGRlYWwgd2l0aCBudW1lcmljYWwgcHJlY2lzaW9uIGlzc3VlcwogICAgfSBlbHNlIHsKICAgICAgZGlzdC5jbHVzdGVyLmNlbGxzIDwtIDAKICAgICAgY2x1c3Rlci5kaWFtZXRlciA8LSAwCiAgICB9CiAgICBkaXN0MmNlbnRyZSA8LSBhcHBseShjZWxsc1ssIGMoIngiLCJ5IiwieiIpXSwgMSwgZnVuY3Rpb24oeCkge2Rpc3QocmJpbmQoeCwgY2x1c3Rlci5jZW50cmUpKX0pCiAgICB2b2x1bWUuY2VsbHMgPC0gY2VsbHNbd2hpY2goZGlzdDJjZW50cmU8PWNsdXN0ZXIuZGlhbWV0ZXIpLCBjKCJ4IiwieSIsInoiKV0gCiAgICBuLmNsdXN0ZXIgPC0gbnJvdyh2b2x1bWUuY2VsbHMpCiAgICAKICAgICMgTnVtYmVyIG9mIHRyYW5zZHVjZWQgY2VsbHMgaW4gdGhlIGNsdXN0ZXIKICAgIG4udHJhbnNkdWNlZC5jbHVzdGVyIDwtIG5yb3coY2x1c3Rlci5jZWxscykKICAKICAgICMgQXZlcmFnZSBwYWlyd2lzZSBkaXN0YW5jZSBiZXR3ZWVuIHRyYW5zZHVjZWQgY2VsbHMgaW4gdGhlIGNsdXN0ZXIKICAgIGF2Zy5kaXN0LmNsdXN0ZXIuY2VsbHMgPC0gbWVhbihkaXN0LmNsdXN0ZXIuY2VsbHMpCiAgICAKICAgICMgQXZlcmFnZSBwYWlyd2lzZSBkaXN0YW5jZSBiZXR3ZWVuIGFsbCBjZWxscyBpbiB0aGUgY2x1c3RlcgogICAgaWYgKG5yb3codm9sdW1lLmNlbGxzKT4xKSB7CiAgICAgIGF2Zy5kaXN0LnZvbHVtZS5jZWxscyA8LSBtZWFuKGRpc3Qodm9sdW1lLmNlbGxzKSkKICAgIH0gZWxzZSB7CiAgICAgIGF2Zy5kaXN0LnZvbHVtZS5jZWxscyA8LSBhdmcuZGlzdC5jbHVzdGVyLmNlbGxzCiAgICB9ICAgIAogIAogICAgIyBOdW1iZXIgb2YgY29udGFjdHMgYmV0d2VlbiB0cmFuc2R1Y2VkIGNlbGxzIGluIHRoZSBjbHVzdGVyCiAgICAjIFR3byBjZWxscyBhcmUgcHJlc3VtZWQgaW4gY29udGFjdCBpZiB0aGV5IGFyZSBsZXNzIHRoYW4gMSBjZWxsIGRpYW1ldGVyICgrIHNkKSBhcGFydCAKICAgIG4uY29udGFjdHMudHJhbnNkdWNlZCA8LSBsZW5ndGgod2hpY2goZGlzdC5jbHVzdGVyLmNlbGxzPD1hdmcuY2VsbC5kaWFtZXRlcisyKnNkLmNlbGwuZGlhbWV0ZXIpKQogICAgCiAgICAjIEZyYWN0aW9uIG9mIHRyYW5zZHVjZWQgY2VsbHMgaW4gdGhlIGNsdXN0ZXIgdm9sdW1lIChpbiBwZXJjZW50KQogICAgZi50cmFuc2R1Y2VkIDwtIDEwMAogICAgaWYgKG4uY2x1c3Rlcj4wKSB7CiAgICAgIGYudHJhbnNkdWNlZCA8LSBuLnRyYW5zZHVjZWQuY2x1c3Rlci9uLmNsdXN0ZXIgKiAxMDAKICAgIH0KICAgIAogICAgIyBEb2VzIHRoZSBjbHVzdGVyIGdpdmUgcmlzZSB0byBhIHR1bW9yID8KICAgIHR1bW9yIDwtIGlmKHN1bShjbHVzdGVyLmNlbGxzJHR1bW9yPjApKSAxIGVsc2UgMAogICAgCiAgICBkZiA8LSBkYXRhLmZyYW1lKGlkID0gaWQsIAogICAgICAgICAgICAgICAgICAgICBudW1iZXIub2YuY2VsbHMuaW4ub3JnYW5vaWQgPSBuLm9yZ2Fub2lkLAogICAgICAgICAgICAgICAgICAgICBvcmdhbm9pZC5jZWxsLmRlbnNpdHkgPSBjZWxsLmRlbnNpdHksCiAgICAgICAgICAgICAgICAgICAgIG51bWJlci5vZi50cmFuc2R1Y2VkLmNlbGxzLmluLm9yZ2Fub2lkID0gbi50cmFuc2R1Y2VkLm9yZ2Fub2lkLCAKICAgICAgICAgICAgICAgICAgICAgbnVtYmVyLm9mLmNlbGxzLmluLmEuY2x1c3RlciA9IG4uY2x1c3RlciwKICAgICAgICAgICAgICAgICAgICAgbnVtYmVyLm9mLnRyYW5zZHVjZWQuY2VsbHMuaW4uYS5jbHVzdGVyID0gbi50cmFuc2R1Y2VkLmNsdXN0ZXIsCiAgICAgICAgICAgICAgICAgICAgIGF2ZXJhZ2UuZGlzdGFuY2UuYmV0d2Vlbi5jZWxscy5pbi5hLmNsdXN0ZXIgPSBhdmcuZGlzdC52b2x1bWUuY2VsbHMsCiAgICAgICAgICAgICAgICAgICAgIGF2ZXJhZ2UuZGlzdGFuY2UuYmV0d2Vlbi50cmFuc2R1Y2VkLmNlbGxzLmluLmEuY2x1c3RlciA9IGF2Zy5kaXN0LmNsdXN0ZXIuY2VsbHMsCiAgICAgICAgICAgICAgICAgICAgIGZyYWN0aW9uLm9mLnRyYW5zZHVjZWQuY2VsbHMuaW4uYS5jbHVzdGVyID0gZi50cmFuc2R1Y2VkLAogICAgICAgICAgICAgICAgICAgICBudW1iZXIub2YuY29udGFjdHMuYmV0d2Vlbi50cmFuc2R1Y2VkLmNlbGxzLmluLmEuY2x1c3RlciA9IG4uY29udGFjdHMudHJhbnNkdWNlZCwKICAgICAgICAgICAgICAgICAgICAgdHVtb3IgPSB0dW1vcikKICAgIAogICAgY2x1c3RlcnMgPC0gcmJpbmQoY2x1c3RlcnMsIGRmKQogICAgCiAgfQogIG9yZy50dW1vciA8LSBpZihzdW0oY2VsbHMkdHVtb3I+MCkpIDEgZWxzZSAwCiAgb3JnYW5vaWQuZmVhdHVyZXNbaSxdIDwtIGMobnJvdyhjZWxscyksIGNlbGwuZGVuc2l0eSwgbiwgb3JnLnR1bW9yKQp9CgpgYGAKCiMjIyMgTG9naXN0aWMgcmVncmVzc2lvbiB3aXRoIG1vZGVsIHNlbGVjdGlvbgpJbiB0aGlzIGFwcHJvYWNoLCB3ZSBidWlsZCBhIG1vZGVsIGZvciBlYWNoIHBvc3NpYmxlIGNvbWJpbmF0aW9uIG9mIGZlYXR1cmVzIChpLmUuIDIqKjkgbW9kZWxzIGZvciA5IGZlYXR1cmVzKSBhbmQgdXNlIHRoZSBBa2Fpa2UgaW5mb3JtYXRpb24gY3JpdGVyaW9uIHRvIHNlbGVjdCB0aGUgYmVzdCBtb2RlbCBhbmQgZXZhbHVhdGUgdGhlIGNvbnRyaWJ1dGlvbiBvZiBmZWF0dXJlcyByZXRhaW5lZCBpbiB0aGlzIG1vZGVsLgpCZWNhdXNlIGl0IGlzIHBvc3NpYmxlIHRoZSBBSUMgaXMgc3ViamVjdCB0byBzbWFsbCB2YXJpYXRpb25zIGFuZCBiZWNhdXNlIG1vZGVscyBhcmUgY29uc2lkZXJlZCBpbmRpc3Rpbmd1aXNoYWJsZSBpZiB0aGUgZGlmZmVyZW5jZSBpbiBBSUMgaXMgbGVzcyB0aGFuIDIsIHdlIGFsc28gbG9vayBhdCB2YXJpYWJsZSBpbXBvcnRhbmNlIG92ZXIgbXVsdGlwbGUvYWxsIG1vZGVscwpgYGB7cn0KbGlicmFyeShnbG11bHRpKQpsaWJyYXJ5KHNqUGxvdCkKCiMgQXV0b21hdGljYWxseSBzZWxlY3QgYmVzdCBtb2RlbChzKQptb2RlbHMgPC0gZ2xtdWx0aSh0dW1vciB+IC4sIGRhdGEgPSBjbHVzdGVyc1ssLTFdLCAjIFJlbW92ZSBJRCBjb2x1bW4KICAgICAgICAgICAgICAgICAgaW50ZXJjZXB0ID0gVFJVRSwKICAgICAgICAgICAgICAgICAgbGV2ZWwgPSAxLCAgICAgICAgICAgICAgICAjIERvbid0IGNvbnNpZGVyIHBhaXJ3aXNlIGludGVyYWN0aW9ucwogICAgICAgICAgICAgICAgICBtZXRob2QgPSAiaCIsICAgICAgICAgICAgICMgRXhwbG9yZSBhbGwgc2V0IG9mIGNhbmRpZGF0ZXMKICAgICAgICAgICAgICAgICAgY3JpdCA9ICJhaWNjIiwgICAgICAgICAgICAjIEFJQyBhcyBjcml0ZXJpYSAod2l0aCBjb3JyZWN0aW9uIGZvciBzbWFsbCBzYW1wbGVzKQogICAgICAgICAgICAgICAgICBjb25mc2V0c2l6ZSA9IDIqKjksICAgICAgICMgTnVtYmVyIG9mIG1vZGVscyB0byBrZWVwLgogICAgICAgICAgICAgICAgICBwbG90dHkgPSBGLCByZXBvcnQgPSBGLCAgICMgTm8gcGxvdCBvciBpbnRlcmltIHJlcG9ydHMKICAgICAgICAgICAgICAgICAgZml0ZnVuY3Rpb24gPSAiZ2xtIiwgICAgICAjIFVzZSBnbG0oKSBmdW5jdGlvbgogICAgICAgICAgICAgICAgICBmYW1pbHkgPSBiaW5vbWlhbCkgICAgICAgICMgYmlub21pYWwgZmFtaWx5IGZvciBsb2dpc3RpYyByZWdyZXNzaW9uCgojIFBsb3QgdGhlIEFJQ2MgdmFsdWVzCiMgVGhlIHJlZCBsaW5lIGluZGljYXRlcyBhIGNvbnZlbnRpb25hbCBjdXQtb2ZmIGZvciBtb2RlbCBzZWxlY3Rpb24KIyAoaS5lLiBsZXNzIHRoYW4gMiBBSUMgdW5pdHMgZnJvbSB0aGUgYmVzdCBtb2RlbCkKcGxvdChtb2RlbHMpCgojIEdldCBtb2RlbC1hdmVyYWdlZCBlc3RpbWF0ZXMgb2YgZmVhdHVyZSB3ZWlnaHRzIGZvciB0aGUgdG9wIG1vZGVscyB3aXRoaW4gMyBJQyB1bml0IG9mIHRoZSBiZXN0CmNvZWYobW9kZWxzLCBzZWxlY3QgPSAzLjApCgojIFBsb3QgcmVsYXRpdmUgaW1wb3J0YW5jZSBvZiB0aGUgZmVhdHVyZXMgdGFraW5nIGFsbCBvZiB0aGUgbW9kZWxzIGludG8gY29uc2lkZXJhdGlvbi4KIyBGb3IgZWFjaCBmZWF0dXJlLCB0aGlzIGlzIHRoZSBzdW0gb2YgdGhlIHdlaWdodHMvcHJvYmFiaWxpdGllcyBmb3IgdGhlIG1vZGVscyBpbiB3aGljaCB0aGUgZmVhdHVyZSBhcHBlYXJzLgojIFRoZSB2ZXJ0aWNhbCByZWQgbGluZSBhdCAuOCBpcyBhbiBvZnRlbiB1c2VkIGN1dG9mZiB0byBkaWZmZXJlbnRpYXRlIGJldHdlZW4gaW1wb3J0YW50IGFuZCBub3QgaW1wb3J0YW50IHZhcmlhYmxlcy4KcGFyKG1hcj1jKDUsMTIsNCwxKSsuMSkgIyBDaGFuZ2UgbGVmdCBtYXJnaW4gc2l6ZSB0byB3cml0ZSBsb25nIHZhcmlhYmxlIG5hbWVzCnBsb3QobW9kZWxzLCB0eXBlID0gInMiLCBsYXMgPSAxKQoKIyAjIFNob3cgc2Vjb25kIGJlc3QgbW9kZWwKIyBwbG90X21vZGVsKG1vZGVsc0BvYmplY3RzW1syXV0sIHNvcnQuZXN0ID0gVFJVRSwgc2hvdy52YWx1ZXMgPSBUUlVFLCB2YWx1ZS5vZmZzZXQgPSAuMywgdmxpbmUuY29sb3IgPSAiZ3JleSIpCiMgcHJpbnQoc3VtbWFyeShtb2RlbHNAb2JqZWN0c1tbMl1dKSkKCiMgR2V0IGJlc3QgbW9kZWwKYmVzdC5maXQgPC0gbW9kZWxzQG9iamVjdHNbWzFdXQpwbG90X21vZGVsKGJlc3QuZml0LCBzb3J0LmVzdCA9IFRSVUUsIHNob3cudmFsdWVzID0gVFJVRSwgdmFsdWUub2Zmc2V0ID0gLjMsIHZsaW5lLmNvbG9yID0gImdyZXkiKQpwcmludChzdW1tYXJ5KGJlc3QuZml0KSkKCgpgYGAKCiMjIyMgTG9naXN0aWMgcmVncmVzc2lvbiB3aXRoIGVsYXN0aWMgbmV0IHJlZ3VsYXJpemF0aW9uClRoaXMgYXBwcm9hY2ggc3RhcnRzIHdpdGggYWxsIGZlYXR1cmVzIGluIHRoZSBtb2RlbCBhbmQgdHJpZXMgdG8gc2V0IHRvIDAgYXMgbWFueSBvZiB0aGUgY29lZmZpY2llbnRzIGFzIHBvc3NpYmxlLgpgYGB7cn0KbGlicmFyeShnbG1uZXQpCgpzZXQuc2VlZCgyMDE4MTIwNCkKCmZlYXR1cmVzIDwtIGFzLm1hdHJpeChzdWJzZXQoY2x1c3RlcnMsIHNlbGVjdCA9IC1jKGlkLCB0dW1vcikpKQojIGFscGhhID0gMSAtLT4gTEFTU08KbW9kZWwgPC0gZ2xtbmV0KGZlYXR1cmVzLCBmYWN0b3IoY2x1c3RlcnMkdHVtb3IpLCBmYW1pbHkgPSAiYmlub21pYWwiLCBhbHBoYSA9IDEpCgojIFBsb3QgZXZvbHV0aW9uIG9mIHRoZSBjb2VmZmljaWVudHMgYXMgYSBmdW5jdGlvbiBvZiByZWd1bGFyaXphdGlvbi4KIyBBIHNtYWxsIEwxIG5vcm0gbWVhbnMgYSBzdHJvbmcgcmVndWxhcml6YXRpb24sIHdpdGggMCBjb3JyZXNwb25kaW5nIHRvIGFuIGVtcHR5IG1vZGVsLgojIEFzIHJlZ3VsYXJpemF0aW9uIGlzIHJlbGF4ZWQgKGluY3JlYXNlIGluIHRoZSBMMSBub3JtKSwgbW9yZSBmZWF0dXJlcyBlbnRlciB0aGUgbW9kZWwuCiMgVGhlcmVmb3JlIG1vcmUgaW1wb3J0YW50IGZlYXR1cmVzIGNhbiBiZSBzZWVuIGFzIGVudGVyaW5nIHRoZSBtb2RlbCBlYXJseS4KcGFyKG1hcj1jKDQuNSw0LjUsMiw4KSkgIyBJbmNyZWFzZSByaWdodCBtYXJnaW4gdG8gd3JpdGUgdmFyaWFibGUgbmFtZXMKcGxvdChtb2RlbCkKY29lZmYucGF0aHMgPC0gY29lZihtb2RlbCkKbGFzdC52YWx1ZXMgPC0gY29lZmYucGF0aHNbLTEsbmNvbChjb2VmZi5wYXRocyldICMgcmVtb3ZlIHRoZSBpbnRlcmNlcHQsIGFuZCBrZWVwIHRoZSBjb2VmZmljaWVudHMgYXQgdGhlIGVuZCBvZiB0aGUgcGF0aApheGlzKDQsIGF0ID0gbGFzdC52YWx1ZXMsIGxpbmUgPSAtLjUsIGxhYmVsID0gbmFtZXMobGFzdC52YWx1ZXMpLCBsYXMgPSAxLCB0aWNrID0gRkFMU0UsIGNleC5heGlzID0gMC41KSAKCiMgR2V0IGZlYXR1cmUgY29lZmZpY2llbnRzIHdpdGggY3Jvc3MtdmFsaWRhdGlvbgptb2RlbCA8LSBjdi5nbG1uZXQoZmVhdHVyZXMsIGZhY3RvcihjbHVzdGVycyR0dW1vciksIGZhbWlseSA9ICJiaW5vbWlhbCIsIHR5cGUgPSAiZGV2aWFuY2UiLCBuZm9sZHMgPSA1KQpwbG90KG1vZGVsKQpwcmludChjb2VmKG1vZGVsLCBzID0gImxhbWJkYS5taW4iKSkKCmBgYAoK
