# Supplementary material for: Tracking cells in epithelial acini by light sheet microscopy reveals proximity effects in breast cancer initiation
Source: eLife. 2020 Jul 21;9:e54066. doi: 10.7554/eLife.54066 (PMC7373425; doi:10.7554/eLife.54066)
Supplement: Transparent reporting form [file elife-54066-transrepform.docx]

***eLife’s* transparent reporting form**

We encourage authors to provide detailed information *within their submission* to facilitate the interpretation and replication of experiments. Authors can upload supporting documentation to indicate the use of appropriate reporting guidelines for health-related research (see [EQUATOR Network](http://www.equator-network.org/%20)), life science research (see the [BioSharing Information Resource](https://biosharing.org/)), or the [ARRIVE guidelines](http://www.plosbiology.org/article/info:doi/10.1371/journal.pbio.1000412) for reporting work involving animal research. Where applicable, authors should refer to any relevant reporting standards documents in this form.

If you have any questions, please consult our Journal Policies and/or contact us: [editorial@elifesciences.org](mailto:editorial@elifesciences.org).

**Sample-size estimation**

- You should state whether an appropriate sample size was computed when the study was being designed
- You should state the statistical method of sample size computation and any required assumptions
- If no explicit power analysis was used, you should describe how you decided what sample (replicate) size (number) to use

Please outline where this information can be found within the submission (e.g., sections or figure legends), or explain why this information doesn’t apply to your submission:

*All experiments involve 3D cell cultures grown from single cells harvested from murine mammary glands. Biological variability is thus accounted for by using different mice with the same genotype for growing acini.*

*Acini in a 3D culture gel are developing from single mammary epithelial cells of the same mouse. Technical variability is thus accounted for by analyzing multiple acinus structures in the same gel.*

*From previous experience, 3 biological replicates and over 5 technical replicates were sufficient to ascertain phenotypes.*

**Replicates**

- You should report how often each experiment was performed
- You should include a definition of biological versus technical replication
- The data obtained should be provided and sufficient information should be provided to indicate the number of independent biological and/or technical replicates
- If you encountered any outliers, you should describe how these were handled
- Criteria for exclusion/inclusion of data should be clearly stated
- High-throughput sequence data should be uploaded before submission, with a private link for reviewers provided (these are available from both GEO and ArrayExpress)

Please outline where this information can be found within the submission (e.g., sections or figure legends), or explain why this information doesn’t apply to your submission:

*All 3D culture experiments involving immunofluorescence staining (followed by confocal microscopy) and long-term light sheet imaging were performed on acini grown from the mammary glands of virgin female mice, between 8-10 weeks old and of the indicated genotype.*

*For each staining experiment, cultures from 3 biological replicates (individual mice) were analyzed at the indicated timepoints. Every 3D culture gel granted 10-15 technical replicates (single acini) for the analysis (Figures 1b, 2a, 2b).*

*For each long-term imaging experiment, cultures from 3 biological replicates were imaged over 3-4 days. Every imaging experiment typically included 6-8 technical replicates (individual acini) imaged in 4D. No full-length (at least 3 day) acinus movies were excluded from the analysis (Figures 3a, 3b). A total of n=20 acini imaged for at least 72 hours have been used for computational feature analysis (Figure 4b).*

*Acini used in long term imaging experiments and their single cell behavior were grouped into binary segments – proliferative and non-proliferative. As such, no “outliers” were encountered.*

*qPCR analysis performed in Figure 1 – figure supplement 1 involves n=2 biological replicates and n=3 technical replicates as indicated in the figure legend.*

**Statistical reporting**

- Statistical analysis methods should be described and justified
- Raw data should be presented in figures whenever informative to do so (typically when N per group is less than 10)
- For each experiment, you should identify the statistical tests used, exact values of N, definitions of center, methods of multiple test correction, and dispersion and precision measures (e.g., mean, median, SD, SEM, confidence intervals; and, for the major substantive results, a measure of effect size (e.g., Pearson's r, Cohen's d)
- Report exact p-values wherever possible alongside the summary statistics and 95% confidence intervals. These should be reported for all key questions and not only when the p-value is less than 0.05.

Please outline where this information can be found within the submission (e.g., sections or figure legends), or explain why this information doesn’t apply to your submission:

*Statistical analysis was performed in Figure 1- figure supplement 1 and the related statistical parameters can be found in the figure legend.*

*Feature analysis and the use of a logistic regression model to assess the probability of tumorigenic outgrowth is described in detail in the Online methods section (specifically lines 675 to 725 of the submitted manuscript); outcome and respective p-values are displayed in Figure 4b, Figure 4b – figure supplement 2.*

(For large datasets, or papers with a very large number of statistical tests, you may upload a single table file with tests, Ns, etc., with reference to sections in the manuscript.)

**Group allocation**

- Indicate how samples were allocated into experimental groups (in the case of clinical studies, please specify allocation to treatment method); if randomization was used, please also state if restricted randomization was applied
- Indicate if masking was used during group allocation, data collection and/or data analysis

Please outline where this information can be found within the submission (e.g., sections or figure legends), or explain why this information doesn’t apply to your submission:

*The experiments consisted of technical and biological replicates. No randomization or group allocation was necessary.*

**Additional data files (“source data”)**

- We encourage you to upload relevant additional data files, such as numerical data that are represented as a graph in a figure, or as a summary table
- Where provided, these should be in the most useful format, and they can be uploaded as “Source data” files linked to a main figure or table
- Include model definition files including the full list of parameters used
- Include code used for data analysis (e.g., R, MatLab)
- Avoid stating that data files are “available upon request”

Please indicate the figures or tables for which source data files have been provided:

*1) Entire image recordings (movies) of time-lapse panels in Figure 3a and 3b (3 Video files in total) have been provided as supplementary movie files.*

*2) We have uploaded the code for the Feature analysis of the nine acinar features described in Figure 4, as source code file “Feature_Analysis.Rmd”. Refer to Supplement File 1 and Online Materials and Methods section for analysis summary.*

*3) We have uploaded the html file describing the source code as Supplementary File 1.*

*4) Three .xlsx files with 20 sheets each, one sheet for each acinus analyzed are provided as Supplementary File 2, 3,4. These contain the x,y,z coordinates for each cell in the respective acinus at the beginning of the SPIM recording(Supplementary File 2) and at the end(Supplementary File 3). Supplementary File 4 contains the "label" for each transduced cell (corresponding to the labels in Supplement File 2) for the acini at the beginning of the SPIM recording. These .xlsx files were input into the source code to carry out the acinus feature analysis described in Figure 4b and Figure 4 – figure supplement 1.*

*4) We have deposited the original imaging data for all acini recorded and analyzed (20 mammary acini) at the BioStudies archive at EMBL-EBI. The data can be accessed confidentially for review using the following key:* <https://www.ebi.ac.uk/biostudies/studies/S-BIAD13?key=d65c53a7-253d-4e4e-82df-d27be3a836f6>

*A total of 390-450 .h5 image files recorder from 2 channels on the microscope are uploaded for each acini (10 minute time intervals). Raw image data from the microscope was cropped to remove empty pixels, binned in x,y (3,3) and converted to 8-bit images using Big Data Processor Fiji Plug in (Tischer, C., Norlin, N., & Pepperkok, R. (2019). "BigDataProcessor; Fiji plugin for visual inspection and processing of big image data.* [*http://doi.org/10.5281/zenodo.2574702*](http://doi.org/10.5281/zenodo.2574702)*.)*

*This data repository also contains video files generated via Imaris for each acinus, showing fluorescence SPIM miscropscopy data (pre-processed raw files available in respective folders) in 2-color 3D projections (mcherry- magenta; GFP- green) for observing visual phenotypes.*
